# Targeted next-generation sequencing of deafness genes in hearing-impaired individuals uncovers informative mutations

**DOI:** 10.1038/gim.2014.65

**Published:** 2014-05-29

**Authors:** Barbara Vona, Tobias Müller, Indrajit Nanda, Cordula Neuner, Michaela A. H. Hofrichter, Jörg Schröder, Oliver Bartsch, Anne Läßig, Annerose Keilmann, Sebastian Schraven, Fabian Kraus, Wafaa Shehata-Dieler, Thomas Haaf

**Affiliations:** 1 Institute of Human Genetics, Julius-Maximilians-Universität Würzburg, Würzburg, Germany; 2 Department of Bioinformatics, Julius-Maximilians-Universität Würzburg, Würzburg, Germany; 3 Institute of Human Genetics, University Medical Centre, Johannes Gutenberg University, Mainz, Germany; 4 Division of Communication Disorders, Department of Otorhinolaryngology, University Medical Centre, Johannes Gutenberg University, Mainz, Germany; 5 Department of Otorhinolaryngology, Plastic, Aesthetic and Reconstructive Head and Neck Surgery, Comprehensive Hearing Center, Julius-Maximilians-Universität Würzburg, Würzburg, Germany.

**Keywords:** deafness gene panel, mutational load, nonsyndromic hearing loss, sensorineural hearing loss, targeted next-generation sequencing

## Abstract

**Purpose:**

Targeted next-generation sequencing provides a remarkable opportunity to identify variants in known disease genes, particularly in extremely heterogeneous disorders such as nonsyndromic hearing loss. The present study attempts to shed light on the complexity of hearing impairment.

**Methods:**

Using one of two next-generation sequencing panels containing either 80 or 129 deafness genes, we screened 30 individuals with nonsyndromic hearing loss (from 23 unrelated families) and analyzed 9 normal-hearing controls.

**Results:**

Overall, we found an average of 3.7 variants (in 80 genes) with deleterious prediction outcome, including a number of novel variants, in individuals with nonsyndromic hearing loss and 1.4 in controls. By next-generation sequencing alone, 12 of 23 (52%) probands were diagnosed with monogenic forms of nonsyndromic hearing loss; one individual displayed a DNA sequence mutation together with a microdeletion. Two (9%) probands have Usher syndrome. In the undiagnosed individuals (10/23; 43%) we detected a significant enrichment of potentially pathogenic variants as compared to controls.

**Conclusion:**

Next-generation sequencing combined with microarrays provides the diagnosis for approximately half of the *GJB2* mutation–negative individuals. Usher syndrome was found to be more frequent in the study cohort than anticipated. The conditions in a proportion of individuals with nonsyndromic hearing loss, particularly in the undiagnosed group, may have been caused or modified by an accumulation of unfavorable variants across multiple genes.

## Introduction

Hereditary hearing loss (HL) is one of the most common birth defects, with an approximate incidence of 1–2 per 1,000 newborns presenting bilateral sensorineural HL at the time of newborn hearing screening. In developed countries, HL stems from both environmental and genetic etiological factors, with the genetic contribution comprising 50–60% of cases.^1,2^

Because of the Mendelian nature of nonsyndromic HL (NSHL), the search for new genes has witnessed profound achievement, particularly in the past decade. NSHL demonstrates extreme genetic heterogeneity, with more than 54 autosomal dominant (deafness, neurosensory, autosomal-dominant (DFNA)), 75 autosomal recessive (deafness, neurosensory, autosomal-recessive (DFNB)), and 5 X-linked (deafness, neurosensory, X-linked (DFNX)) loci with 27, 44, and 3 causative genes, respectively, identified to date (http://hereditaryhearingloss.org). A fraction of these genes have been associated with both dominant and recessive HL. Furthermore, mitochondrial mutations can also underlie NSHL. Next-generation sequencing (NGS) technologies are causing a shift in how clinical geneticists and medical researchers investigate genetic disorders^3^ and provide powerful application not only to molecular diagnostics but also to the discovery of new genes and further characterization of already-known disease-associated genes.^4,5,6^ Of particular interest to clinicians is target capture NGS involving a subset of disease-relevant genes in the form of gene panels that accommodate sequencing of dozens or hundreds of genes in parallel, with a clear advantage over conventional polymerase chain reaction–based Sanger sequencing approaches by achieving faster results at a fraction of the cost.^7^

A further application of NGS is learning the variation landscape of the minor allele load on a gene-by-gene, exome-wide, or genome-wide basis in affected and unaffected individuals. Understanding the concept of mutational load in human disorders will provide insight into the potential role of rare nonsynonymous single-nucleotide polymorphisms (SNPs), their maintenance throughout human evolution, and their predication underlying human disease. By shifting emphasis away from individual frequencies of deleterious variants toward cumulative frequencies, explanations for common disorders with complex inheritance become plausible.^8^

In this study, we used one of two gene panels consisting of either 80 or 129 deafness genes using NGS to detect damaging variants in 30 individuals from 23 unrelated families with a broad range of HL onset and severity, with an initial goal of HL diagnostics. The members of the remaining undiagnosed cohort (14 probands from 10 unrelated families) were carefully compared against 9 normal-hearing controls for enrichment of deleterious variants.

## Materials and Methods

### Case evaluation, classification, and controls

Thirty individuals with hearing impairment were recruited over a number of years from Würzburg and Mainz, Germany, for targeted deafness gene sequencing after genetic counseling was initiated. All of the probands except one (R5) had been prescreened by conventional Sanger sequencing for mutations in *GJB2*. All parents and participants provided informed written consent. This study was approved by the ethics committee of the University of Würzburg.

Upon diagnosis of HL, patients routinely undergo kidney and thyroid sonography, urinalysis, electrocardiogram, neurological examination, blood profile analysis, and serological examination for infectious disease, as well as ophthalmological examination and magnetic resonance imaging of the brain, inner ear, and temporal bones for the assessment of HL in conjunction with a syndrome. Clinical test results, age of onset, and age of enrollment are summarized in **Supplementary Table S1** online. Pure-tone audiometry and auditory brainstem response were used to assess degree and progression of HL. The following guideline was used to determine severity of HL: 0–20 dB, normal; 20–40 dB, mild; 40–55 dB, moderate; 55–70 dB, moderately severe; 70–90 dB, severe; and >90 dB, profound. Seven of the 30 individuals were family members of affected probands who were included to aid with analysis but not considered for statistics and success rate calculation. When possible, additional family members were also recruited for follow-up cosegregation analysis.

Seventeen of the 23 probands had prelingual HL, which is either present at birth or begins before the age of 5 in the critical time interval for language acquisition. Six individuals had postlingual HL with onset between age 6 and 10 years. From pedigree analysis and familial information we were able to characterize hearing impairment types into three subgroups: dominant (two or more generations affected or mutations detected in genes conferring dominant HL without opportunity for cosegregation analysis; represented by families D1 through D8), recessive (parents are normal hearing, possible consanguinity known; indicated by families R1 through R5), and undiagnosed (which could be consistent with dominant or recessive HL, but based on lack of familial involvement, inheritance category was unconfirmed; as observed in families U1 through U10). In total, we had 8 dominant, 5 recessive, and 10 undiagnosed individuals. The majority of our probands were of European descent, except for D7 and U5, who are Turkish; and R2 and U8, who are of Arab ethnicity. We also included nine unrelated healthy controls with normal hearing and without a family history of HL in our study to investigate the prevalence of pathogenic variants in subjectively normal-hearing individuals and to aid variant filtering.

### Microarray screen

For the exclusion of pathogenic copy-number variation (CNV) in the genome of all hearing-impaired individuals before undergoing target enrichment sequencing, we performed either a SNP array or array comparative genomic hybridization using genomic DNA prepared from peripheral blood by a standard salt extraction method. SNP array CNV detection was performed with an Illumina Omni1-Quad v1.0 chip (Illumina, San Diego, CA) according to the manufacturer's specifications. Array data were analyzed using GenomeStudio version 2011.1 (Illumina) and the QuantiSNP 2.2 copy-number detection algorithm.^9^ Array comparative genomic hybridization was performed using a Roche NimbleGen CGX v1 315K array (Roche NimbleGen, Madison, WI) per manufacturer's recommendations using healthy pooled male and female reference DNA (Promega, Madison, WI), and arrays were analyzed using Genoglyphix software (Signature Genomics, Spokane, WA).

### Target enrichment sequencing, alignment, and variant detection

Genomic DNA from 30 individuals with hearing impairment and 9 normal-hearing individuals was subjected to one of two possible gene panels containing either 80 or 129 genes that are listed in **Supplementary Table S2** online. Both panels shared the same 80 genes, with the 129 gene panel containing additional genes. These panels included NSHL genes with a DFN locus annotation and syndromic HL genes, as well as a limited number of strong candidate HL genes (i.e., from animal experiments). Exome capture and NGS on a HiSeq2000 (Illumina) were performed by Otogenetics Corporation (Norcross, GA). A total of 5 µg genomic DNA at a concentration of 20–500 ng/µl in Tris/EDTA was used as input material for NimbleGen capture methods to generate 2 × 100 paired-end reads. High-quality sequence reads were mapped to the human genome reference (NCBI build 37, hg19), as well as to the reference sequences of the targeted genes in each of the panels using DNAnexus cloud-based data analysis (Mountain View, CA) for variant calling.

Because we did not want to risk losing variants impacting splice sites, pathogenic dbSNP (https://www.ncbi.nlm.nih.gov/SNP) entries, or synonymous variants potentially affecting splice sites, we filtered data conservatively in three areas: (i) mean depth and read counts ≥10; (ii) removal of 3′UTR, 5′UTR, downstream, upstream, and noncoding exon transcript variants; and (iii) removal of non–coding change types. We then referenced dbSNP, the Exome Sequencing Project (http://evs.gs.washington.edu/EVS), and the 1000 Genomes Project (http://browser.1000genomes.org/index.html) to screen rare variants with minor allele frequencies residing around or under 1% of available population frequency data. SIFT,^10^ PolyPhen-2,^11^ MutationTaster,^12^ and Alamut (Interactive Biosoftware, Rouen, France) predicted the consequences of an identified amino acid substitution on protein structure/function and pathogenic potential, and rapidly assessed nucleotide and amino acid conservation, potential protein domain involvement, and nucleotide variation impact on splice site. The Human Gene Mutation Database^13^ was also used to determine whether variants were novel or already associated with a phenotype. As a final step, these variants were screened against the control group and were removed unless already established as a deafness-associated damaging mutation. When potentially pathogenic variants were detected, familial cosegregation analysis followed, if possible, and comparisons between proband and published audiogram and clinical data to substantiate which variants likely underlie HL in the affected individual.

### Sanger sequencing

**Figure 1:**
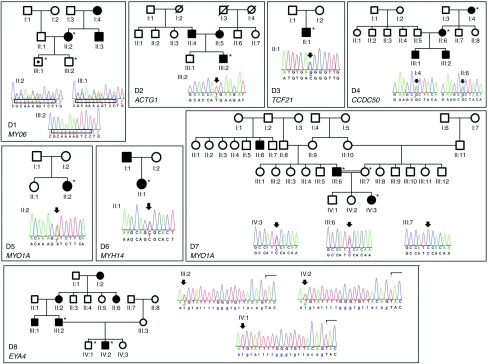
Pedigrees and sequence chromatograms of the autosomal dominant families D1 through D8. Asterisks denote those who were available for sequencing. All mutations are heterozygous. (D1) *MYO6* c.884_893delGCAAAAGTCC (p.Arg295Leufs*13). The deleted sequence under segregation analysis is boxed. The affected index patient (II:2) transmitted the frameshift mutation to one of her two sons (III:1), who was enrolled before the typical age of onset for DFNA22 and is not yet affected. (D2) *ACTG1* c.974T>A (p.Met325Lys). (D3) *TCF21* c.63C>G (p.Asp21Glu). (D4) *CCDC50* c.227G>A (p.Arg76His). (D5) *MYO1A* c.2032A>T (p.Ile678Phe). (D6) *MYH14* c.5008C>T (p.Arg1670Cys). (D7) *MYO1A* c.2390C>T (p.Ser797Phe). (D8) *EYA4* c.1341-19T>A predicted 3′ splice site mutation.

**Figure 2:**
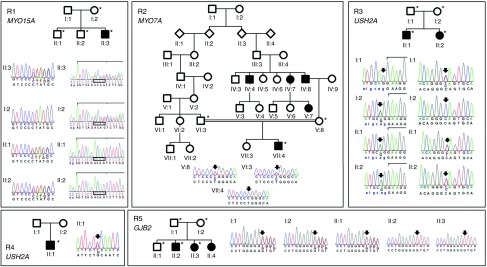
Pedigrees and sequence chromatograms of the autosomal recessive families R1 through R5. *Those who were available for sequencing. (R1) Compound heterozygous *MYO15A* c.1137delC (p.Tyr380Metfs*65) (left) and c.7124_7127delACAG (p.Asp2375Valfs*29) (right) mutations. The deleted sequence under segregation analysis is boxed. (R2) Homozygous *MYO7A* c.3935T>C (p.Leu1312Pro) mutation in a consanguineous family. (R3) Compound heterozygous *USH2A* c.1841-2A>G (left) and c.2440C>T (p.Gln814*) (right) mutations. (R4) Heterozygous *USH2A* c.2276G>T (p.Cys759Phe). (R5) Homozygous *GJB2* c.35delG (p.Gly12Valfs*2).

Candidate variations that remained after filtering were amplified by polymerase chain reaction using primer pairs designed from Primer3 software^14^ for validation. We sequenced all the control variants and damaging mutations shown in **Figures 1** and **2**, as well as additional case variants with less than 50-fold coverage. Primer sequences are available upon request. Polymerase chain reaction products were bidirectionally sequenced with an ABI 3130xl 16-capillary sequencer (Life Technologies, Carlsbad, CA). Sequence reactions were completed with a 5× sequencing buffer and BigDye Terminator (Applied Biosystems, Life Technologies). DNA sequence analysis was performed using Gensearch software (Phenosystems, Lillois Witterzee, Belgium).

### Statistical analysis

Because there were two different panel types in this study, we excluded all genes from the 129-gene panel that were not included in the 80 gene panel. On the basis of these 80 common genes, we analyzed variant distribution. The pairwise Wilcoxon test followed by a Benjamini–Hochberg multiple testing correction was used to determine whether there was a significant difference in the number of variants in the control versus case groups. Multidimensional scaling plots were generated to analyze the gene variant distribution patterns between the undiagnosed and control groups using the statistical framework R (http://www.R-project.org) and the Vegan statistical package.^15^

## Results

### HL and clinical summaries

Audiometric information from the 23 probands revealed a spectrum of severity: Three each had mild and severe HL, respectively, four presented moderate HL, nine had moderately severe HL, and four had profound HL. With one exception (proband U2), the individuals we include have no indication of syndromic background. The Usher syndrome probands disclosed are currently younger than the age of onset for retinitis pigmentosa, which is why we do not currently consider these individuals as syndromic. The most common clinical indication was speech delay, which was present in seven of the probands (D1, D3, R3, R5, U2, U5, and U6), but this is a common occurrence in children with HL, because hearing and speaking are complementary processes. A complete summary of clinical indications and audiograms from available family members is included in **Supplementary Table S1** online.

### Variant analysis

With one notable exception, our probands did not exhibit pathogenic CNVs in the microarray screen. Using a SNP array, the index case of family R4 presented a heterozygous deletion in *USH2A* spanning exons 58–64. This deletion was validated with quantitative real-time polymerase chain reaction in exons 61, 63, and 64 (data not shown).

Targeted deafness gene sequencing of 30 HL individuals (from 23 unrelated families) and 9 normal-hearing controls was performed with one of two panel types consisting of known and suspected HL genes. Twenty-two of 30 individuals (16 of 23 index probands) and 8 of 9 controls were sequenced with the 80-gene panel, and 8 individuals (7 probands and 1 control) were sequenced with the 129-gene panel. The 80-gene panel produced 222.8 kb of targeted sequence, covering 1,258 exons and flanking sequence, and yielded an average of 8.2 ± 1.5 million reads per sample, with approximately 86% mapping to the targeted regions. The average mean depth for the targeted regions was 311.8 ± 86.3; 98.4 ± 2.9% of the exons had a coverage ≥10 reads. The 129-gene panel achieved a total of 313.0 kb of targeted sequence, covering 1,902 exons and flanking sequence. An average of 6.8 ± 0.5 million reads per sample were acquired, with approximately 88% mapping to their targets. The average mean depth for the targeted regions was 246.2 ± 14.9; 98.7 ± 0.1% of the covered exons had ≥10 reads. The run statistics from both panel types per individual are presented in **Supplementary Table S3** online. Missed or low-coverage exons were shared in common among samples.

Analysis of both panel types yielded a total of 89 variants in probands and 14 variants in controls (**Supplementary Table S4** online). The affected individuals had a total of 68 missense, 10 frameshift, 3 indel, 5 nonsense, and 3 splice variants. Controls had 11 missense, 1 frameshift, and 2 indel variants.

### Variant spectrum and diagnosed individuals

Applying conservative filtering strategies to the genes common in both panels, 42 of the 80 target genes did not show a single pathogenic variant in 23 probands and 9 controls. Fourteen genes (*ACTG1*, *COL9A3*, *EYA4*, *GATA3*, *KCNJ10*, *LHFPL5*, *MARVELD2*, *MYO1F*, *MYO3A*, *MYO6*, *OTOA*, *TCF21, TMC1*, and *TMIE*) displayed a single variant, six (*ERCC2*, *ESPN*, *OTOR*, *TMPRSS5*, *USH1C*, and *WSF1*) two variants, seven (*GJB3*, *DSPP*, *MYH9*, *MYO1C*, *PCDH15*, *SPINK5*, and *TECTA*) three variants, seven (*CCDC50*, *CDH23*, *GJB2*, *MYO1A*, *MYO15A*, *SLC26A4*, and *TRIOBP*) four variants, three (*GJB4*, *MYO7A*, and *OTOF*) five variants, and one (*MYH14*) seven variants that met the criteria for potential pathogenicity, evolutionary conservation, and additional filtering criteria such as depth and quality (**Supplementary Table S4** online). A correspondence analysis of the identified variants in the entire data set with 23 probands and 9 controls did not reveal any clustering; in particular, there was no split between affected individuals and controls (data not shown). In this context, it is important to emphasize that all these potentially pathogenic variants represent in silico predictions and usually additional information is needed to identify the disease-causing mutation(s) in a particular case and family. For example, improper segregation of a variant in a dominant family or detection of the same variant in a recessive family or control clearly argues against its pathogenicity. In three probands, we found two damaging variants in a gene conferring recessive HL, that is, in *GPR98* and twice in *OTOF*, but both were inherited on the same allele from a normal-hearing parent. Also, if clinical features and audiograms were not in agreement with the typical HL of a mutated candidate gene, the individual remained in the undiagnosed group.

In 8 of the 23 probands, targeted NGS identified a pathogenic mutation in a gene associated with dominant HL (*ACTG1*, *CCDC50*, *EYA4*, *MYH14*, *MYO6*, *TCF21*, and twice in *MYO1A*). **Table 1** describes the pathogenic variants, with characteristic hearing impairment for each variant. All pathogenic variants were confirmed by Sanger sequencing. The pedigrees of D1, D2, D4, D6, D7, and D8 were consistent with dominant HL (**Figure 1**). Segregation of the mutation with HL could be analyzed in families D1, D4, D7, and D8. In family D2, only the affected child was available for analysis, but given that both parents are hearing impaired, it is likely that one of them has this mutation as well. To our knowledge, D3 and D5 had normal-hearing parents and no family history of HL, suggesting de novo mutation and/or reduced penetrance. However, in each case, clinical information and audiograms were in agreement with typical HL for the affected genes (**Table 1**; **Supplementary Table S1** online) and the mutations occurred in highly conserved amino acids or were predicted to affect gene splicing.

Five probands presented homozygous or compound heterozygous mutations in a gene resulting in recessive HL (*MYO15A*, *MYO7A*, *GJB2*, and twice in *USH2A*) (**Table 1**). The pedigrees were consistent with recessive HL (**Figure 2**). Interestingly, 2 of the 23 probands were referred to our clinics with NSHL but were diagnosed with a mild form of Usher syndrome (type 2A). Neither of the patients had signs of retinitis pigmentosa at the time of diagnosis. Individual R3 and his affected sister were compound heterozygous for a splice site and a nonsense mutation, whereas individual R4 displayed a microdeletion (of exons 58–64) in combination with a missense mutation (**Table 1**). Notably, individual R5, who had been prescreened for mutations in *OTOF* because of suspected auditory neuropathy, was homozygous for the classic c.35delG mutation in *GJB2*.

### Undiagnosed individuals and controls

**Figure 3:**
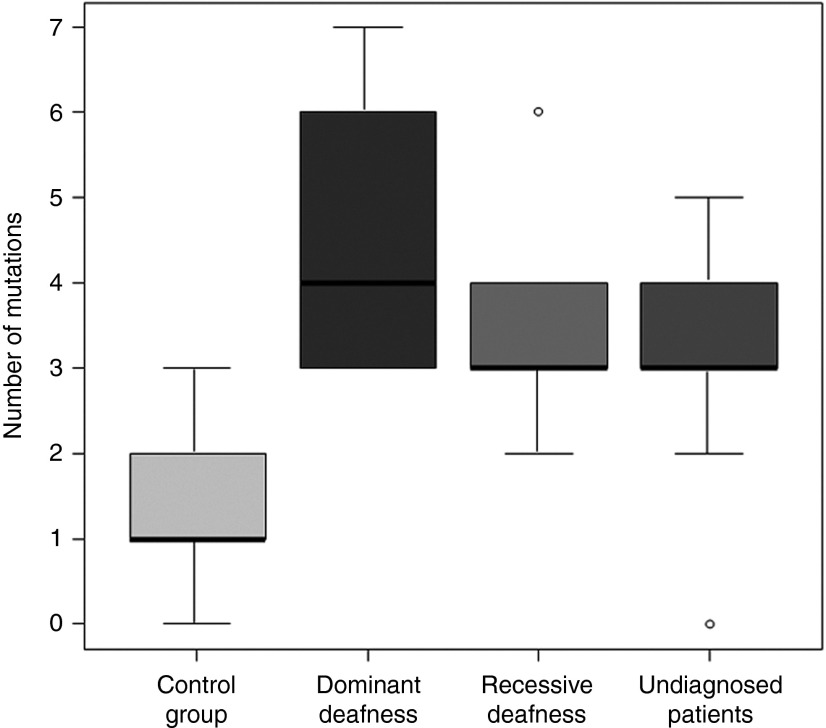
The distribution of variants in the case and control groups among 80 deafness genes. The median number of variants in controls is 1.0, whereas the median number is 4.0 in the dominant group, and 3.0 each in the recessive and undiagnosed groups. This represents a significantly higher number of variants in the case groups as compared to the controls.

Considering only the 80 genes that were screened in all individuals, we detected an average of 4.5 (36/8) potentially damaging variants in probands with dominant HL, 3.6 (18/5) in individuals with recessive HL, 3.0 (30/10) in the undiagnosed group, and 1.4 (13/9) in controls (**Supplementary Table S5** online). The median number of variants was 4 for individuals with dominant HL, 3 each for the recessive and undiagnosed groups, and 1 for controls (**Figure 3**). Pairwise Wilcoxon tests with multiple testing correction revealed significant differences between probands and controls (dominant group versus control, *P* = 0.003; recessive group versus control, *P* = 0.01; and undiagnosed group versus control, *P* = 0.01) but not between different case groups.

One individual from the undiagnosed group and two controls did not display any variant at all. Most (8 of 10; 80%) undiagnosed probands had three or more potentially pathogenic rare variants, whereas most controls (5 of 9; 56%) had fewer than two (**Supplementary Table S5** online). In the control group, we detected damaging variants in *GJB3*, *GJB4*, *MYO1C*, *MYO1F*, *MYO7A*, *PCDH15*, *TMC1*, *TRIOBP*, and *WFS1*, as well as two variants each in *CDH23* and *SPINK5* (**Supplementary Table S4** online). Only one of these variants was in *WFS1*, a gene responsible for dominant HL, and all variants in genes responsible for recessive HL were heterozygous. The individual with the *WSF1* variant describes having episodes of tinnitus when under stress but does not report hearing impairment. A multidimensional scaling analysis^16^ of the undiagnosed and control groups revealed a clustering of primarily the control group near zero and an extensive heterogeneity of probands from the undiagnosed group (**Supplementary Figure S1** online). In other words, individuals from the undiagnosed group show a large variety of different variants, resulting in the extensive heterogeneity in the multidimensional scaling plot. The controls show only a few variants, resulting in a much higher similarity of these individuals and a homogeneous cluster around zero.

## Discussion

### Enrichment of deleterious variants in HL individuals

Studies investigating heterogeneous sensorineural disorders such as intellectual disability and macular degeneration have uncovered the complex variation landscape underlying these phenotypes and detected an accumulation of rare deleterious variants in probands versus controls.^17,18,19^ Consistent with several studies suggesting digenic inheritance of HL,^20,21^ the concept of a mutational load, whereby an excess of deleterious variants scattered across multiple genes^22^ impedes the proper functioning of auditory processes, is an interesting perspective on a typically Mendelian disorder. The enormous complexity of the auditory system suggests elaborate gene interactions may render it vulnerable to accumulation of deleterious variants otherwise tolerable in the context of a neutral genetic environment. Because the majority of missense substitutions with a frequency <1% are deleterious in humans, low allele frequency alone can serve as a predictor of functional significance.^23^ Furthermore, the number of affected genes harboring these rare, deleterious variants could also impact phenotypic consequence.

Evolutionary genetic models predict a cumulative effect of rare, possibly pathogenic, variants scattered across the genome increasing susceptibility to disorders.^23^ Our observation that individuals in the undiagnosed group harbor significantly more damaging variants in HL genes than controls supports this hypothesis. We propose a polygenic or multifactorial form of inheritance in the undiagnosed group, whereby affected genes in combination with other adverse genetic and/or environmental factors may exceed a critical threshold for phenotypic manifestation. However, we cannot exclude the possibility that the increased number of deleterious variants in probands is coincidental. Given the extensive genetic heterogeneity of HL and high marriage rate among hearing-impaired individuals, it is expected that variants accumulate in certain families. In addition, we cannot exclude the possibility that HL in the undiagnosed group is due to monogenic forms of deafness caused by highly penetrant variants in novel genes. Follow-up whole exome sequencing of these individuals could provide answers to this question.

### Application of targeted NGS in routine diagnostics

The great heterogeneity comprising NSHL undoubtedly contributes to molecular diagnostic challenges. In the pre-NGS era, the identification of damaging mutations was dependent on labor- and cost-intensive Sanger sequencing. Routine screening is typically initiated with *GJB2* analysis because 30–40% of NSHL probands with European ancestry have mutations in this gene.^1^ Unless additional clinical symptoms hint at specific genes (i.e., goiter suggesting *SLC26A4* or auditory neuropathy suggesting *OTOF*), the vast majority of *GJB2* mutation–negative probands remain without genetic diagnoses. The development and optimization of NGS gene panels expand the spectrum of disease-relevant genes simultaneously screened in affected individuals with the potential to translate into better case outcomes and support when rare pathogenic mutations are liable.

Through targeted NGS, the most likely causative gene mutations in eight dominant and five recessive individuals were detected, for a success rate of 13 (57%) of 23 probands. Two (9%) individuals displayed compound heterozygous mutations in the *USH2A* gene, which is a higher frequency rate compared to that of a previous study^24^ reporting 11% of *GJB2* mutation–negative children with HL carrying single Usher syndrome mutations. An early diagnosis of Usher syndrome may benefit these children to delay vision loss with basic interventions such as adhering to certain diets and lifestyles,^25^ as well as using eye ultraviolet protection with sunglasses^26^ to slow photoreceptor degeneration. Early diagnosis is especially relevant in our Usher syndrome probands because they are below the age of onset for vision loss. Although it is undeniably important for familial cosegregation analysis for accurate and definitive variant interpretation, we could not always obtain familial DNA. More specifically, individual D3 presented characteristic flat audiometric thresholds for the *TCF21* c.63C>T mutation. Furthermore, this mutation is associated with adult-onset cardiomyopathy, but because he is a child, periodic cardiac monitoring is recommended to detect early signs of dysfunction.^27,28^ Similarly, individual D5 has an audiometric profile with high-frequency HL. Secondary to this audiometric hallmark, *MYO1A* is a gene with variable penetrance.^29^ Furthermore, this variant resides in a myosin motor domain. The *EYA4* variant in family D8 creates a stronger mutated 3′ splice acceptor position as compared with the wild type based on four splice in silico predictor programs. We consider the described mutations (**Table 1**) as diagnostic benchmarks for HL characterization and clarification. In agreement with the success rate of previous studies,^30,31^ our diagnostic yield supports application of this technique for routine diagnostics. However, to enhance the diagnostic potential of NGS, deeper knowledge about population frequencies and pathogenicity of sequence variants is required.

The potential to correct clinical misdiagnosis by broadly screening a predefined gene panel has been previously demonstrated in isolated individuals using exome sequencing without family pedigree information available.^32^ We detected a common *GJB2* c.35delG homozygous mutation in the available affected members of family R5 with profound HL and suspected auditory neuropathy. At the time of clinical evaluation, haplotype analysis was compatible with *OTOF* mutation; however, *OTOF* was negative for mutations and no further sequencing was completed until inclusion in this study. The proband in family R4 was previously included in CNV analysis and presented a heterozygous deletion of exons 58–64 in the 72-exon gene, *USH2A*. Sanger sequencing of this gene for the detection of a second mutation would have been a time- and cost-intensive procedure; however, the NGS panel provided rapid insight into a second *USH2A* mutation.

Because *GJB2* is a single-exon gene accounting for a disproportionate number of HL cases, Sanger sequencing is still recommended for first-line diagnostics. Recent studies^33,34^ showed that besides *GJB2* (DFNB1), *STRC* (DFNB16) is a major contributor to congenital HL, particularly in children with mild to moderate high-frequency HL. A pseudogene with 99.6% coding sequence identity makes it impossible to rely on NGS for *STRC* screening, and a Sanger sequencing protocol excluding the pseudogene is recommended.^34^ We propose targeted NGS deafness gene screening in the remaining undiagnosed individuals. Because CNVs in not only *STRC* but also other deafness genes may significantly contribute to the mutational load, targeted NGS is most powerful in combination with microarray analysis.

### Conclusions

Although a major limitation of our study was the small sample size, we used conservative statistics to avoid overstating our findings. Recent studies have only begun discovering genetic complexities unknown before the advent of NGS technologies. It is noteworthy that all 13 probands diagnosed with a monogenic form of deafness exhibited additional pathogenic variants in other HL genes. It is tempting to speculate that these additional variants have a modifying phenotypic effect, explaining variability in age of onset and progression. As NGS becomes an increasingly conventional method for approaching the genotype–phenotype puzzle, more comprehensive surveys in the future will help elucidate the complexities of HL.

## Disclosure

The authors declare no conflict of interest.

## Figures and Tables

**Table 1:**
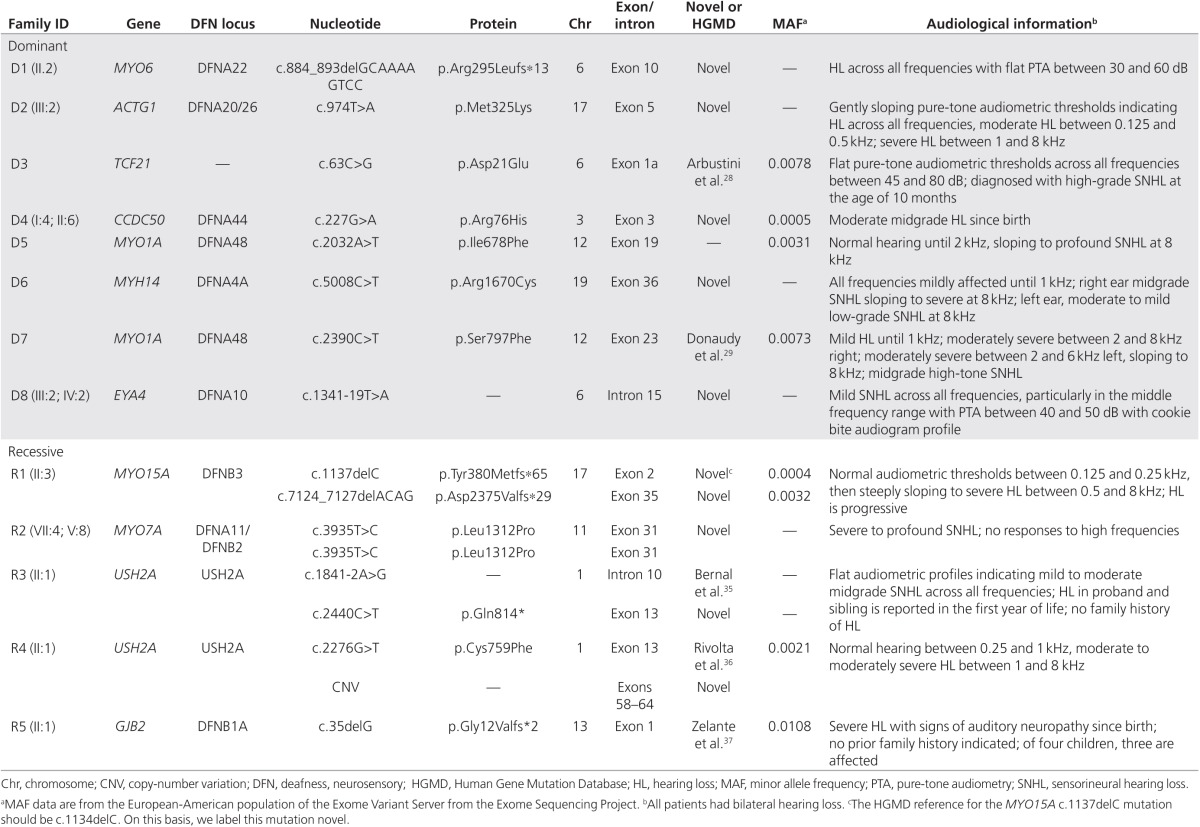
Clinical descriptions and characteristic hearing loss of each dominant or recessive hearing loss patient
