# Supplementary material for: Targeted next-generation sequencing of deafness genes in hearing-impaired individuals uncovers informative mutations
Source: Genet Med. 2014 May 29;16(12):945–53. doi: 10.1038/gim.2014.65 (PMC4262760; doi:10.1038/gim.2014.65)
Supplement: Supplementary Figure S1 [file gim201465x1.doc]

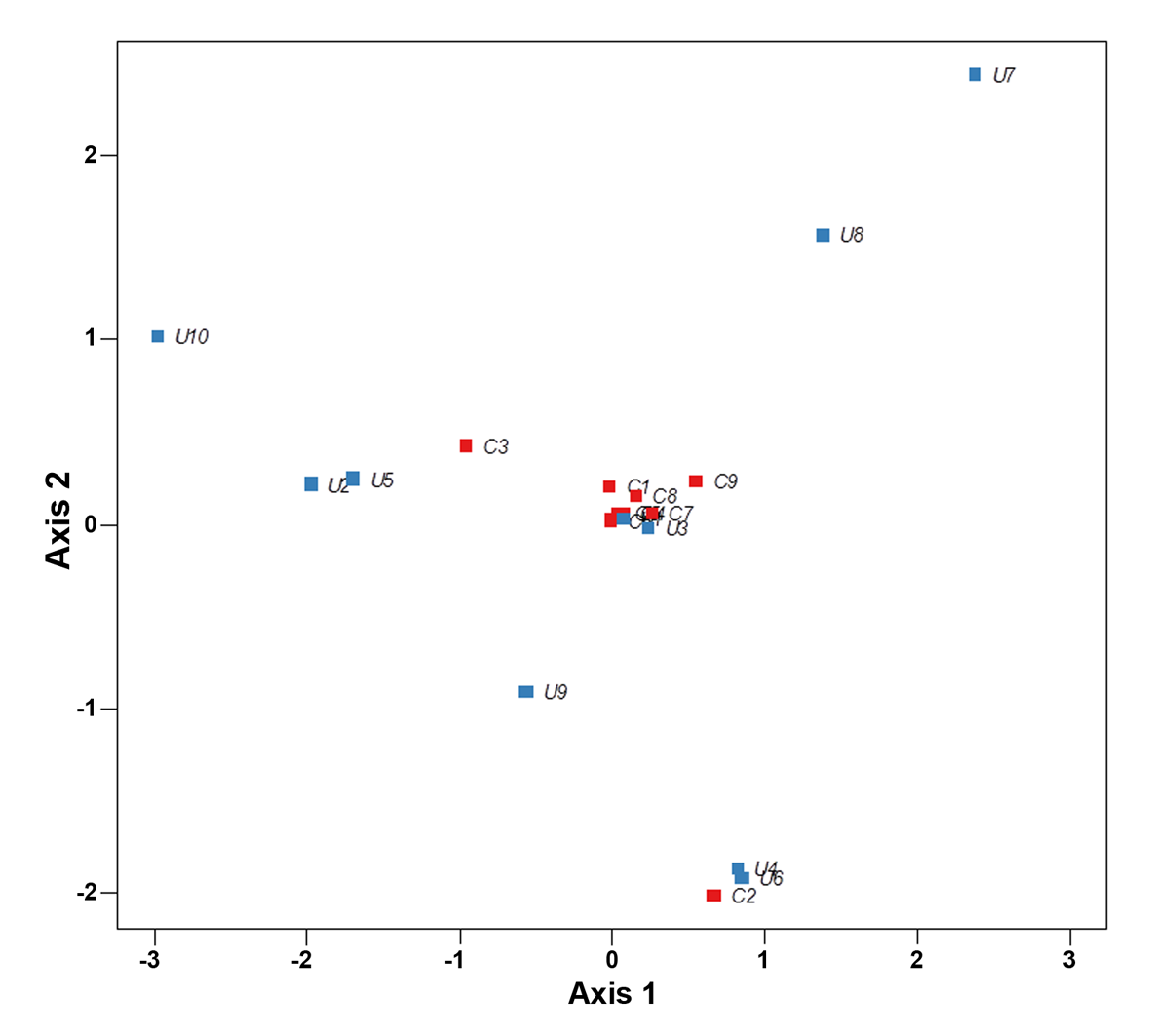


**Figure S1.** Multi-dimensional-scaling plot depicting distribution patterns between control (C) and unsolved (U) groups.
