# Supplementary material for: Targeted next-generation sequencing of deafness genes in hearing-impaired individuals uncovers informative mutations
Source: Genet Med. 2014 May 29;16(12):945–53. doi: 10.1038/gim.2014.65 (PMC4262760; doi:10.1038/gim.2014.65)
Supplement: Supplementary Table S1 [file gim201465x2.doc]

**Table S1** Clinical information from each proband.

| **Case** | **Age of onset (in years)** | **Age at enrollment** | **Clinical Testing** | | | | | **Other clinical information/**  **audiograms from additional family members** |
| --- | --- | --- | --- | --- | --- | --- | --- | --- |
|  |  |  | **Kidney** | **Ophthalmological** | **Thyroid** | **ECG** | **MRI ( inner ear/ temporal bone)** |  |
| D1 | 1 | 1 | Normal | Normal | Normal | Normal | Normal | Speech delay; mother has HL across all frequencies with flat PTA between 30 and 60 dB |
| D2 | At birth | 23 | Normal | Normal | Normal | Normal | Normal | Father is completely deaf; mother has profound HL |
| D3 | 1 | 10 | Normal | Normal | Normal | Normal | Normal | Behavioural problems; ADHD; severe speech delay; intermodular perceptual disturbance (cognition and fine motor function); no evidence of cardiac phenotype but proband is recommended for regular cardiac evaluation |
| D4 | At birth | 44 | Normal | Normal | Normal | Normal | Normal | Grandmother was deaf since birth and mother has profound HL since birth |
| D5 | 7 | 11 | Normal | Normal | Normal | Normal | Normal | Head trauma (right mastoid fracture at one year of age); hearing test was normal at time of fracture |
| D6 | 6 | 14 | Normal | Optic neuritis at five years of age | Normal | Normal | Normal | Otosclerosis; keratosis pilaris; alopecia; irregular EEG waveforms since childhood; father’s audiogram indicates HL with profile similar to his daughter |
| D7 | 5 | 10 | Normal | Normal | Normal | Normal | Normal | Parents are first degree cousins; father’s audiogram also sloping in higher frequencies |
| D8 | 6 | 7 | Normal | Normal | Normal | Normal | Normal | Father has similar cookie bite audiogram profile |
| R1 | 3 | 5 | Normal | Normal | Normal | Normal | Normal | Two brothers tested with normal audiogram results; HL was initially thought to be due to one of three reasons: (1) post-partum hypoxic event, (2) acute vasculitis, or (3) herpes infection at six months of age, but the *MYO15A* segregating mutation challenges these hypotheses |
| R2 | 1-2 | 2 | Normal | Normal | Normal | Normal | Normal | The proband is from a consanguineous union; nuclear family has no indication of HL but has a family history of severe HL |
| R3 | 1-2 | 1 | Normal | Normal | Normal | Normal | Normal | Speech delay, orofacial sensorimotor muscle coordination difficulties, but responding well to phonetic training |
| R4 | 3 | 3 | Normal | Normal, but wears glasses since four years of age | Normal | Normal | Normal | N.a. |
| R5 | At birth | 4 | Normal | Normal | Normal | Normal | Normal | Speech delay |
| U1 | At birth | 3 | Normal | Eyes lasered (retinopathy of prematurity), wears glasses | Normal | Normal | Normal | Delivered 14 weeks prematurely with patent ductus arteriosus; hernia surgery was performed at one year of age |
| U2 | At birth | 6 | Normal | Normal | Normal | Normal | Normal | Failure to thrive, developmental delay, small and low set ears, small hands and feet, motor developmental delay, scoliosis, obstructive sleep apnea, narrowing of tympanic cavity, speech delay, dental malocclusion |
| U3 | 7 | 7 | Normal | Normal | Normal | Normal | Normal | Sudden HL, incomplete simple syndactyly of the second and third proximal phalanges |
| U4 | 5 | 47 | Normal | Normal | Normal | Normal | Normal | N.a. |
| U5 | 1 | 2 | Normal | Normal | Normal | Normal | Normal | Speech delay, oral motor hypotonia, dysphagia, born four weeks prematurely, audiogram from mother was normal |
| U6 | 6 | 7 | Normal | Normal | Normal | Normal | Normal | Speech delay, born five weeks prematurely, audiograms from two other brothers shows one with normal hearing and one with HL |
| U7 | 5 | 6 | Normal | Normal | Normal | Normal | Normal | Grandfather has HL since childhood |
| U8 | 4 | 4 | Normal | Normal | Normal | Normal | Normal | Parents are first degree cousins |
| U9 | 5 | 11 | Normal | Normal | Normal | Normal | Normal | Father has suspected noise exposure HL |
| U10 | 5 | 41 | Normal | Normal | Normal | Normal | Normal | Audiogram of daughter indicates mild HL at the age of six years |

Abbreviations: ADHD, attention deficit hyperactivity disorder; EEG, electrocardiogram; EEG, electroencephalogram; HL, hearing loss; MRI, magnetic resonance imaging; N.a., not available; PTA, pure tone audiometry
