# Supplementary material for: Targeted next-generation sequencing of deafness genes in hearing-impaired individuals uncovers informative mutations
Source: Genet Med. 2014 May 29;16(12):945–53. doi: 10.1038/gim.2014.65 (PMC4262760; doi:10.1038/gim.2014.65)
Supplement: Supplementary Table S2 [file gim201465x3.doc]

**Table S2** Analyzed genes

| **Gene symbol** | **Gene description** | **Deafness locus** | **Chromo-some** | **mRNA length** | **OMIM** | **Reference** |
| --- | --- | --- | --- | --- | --- | --- |
| **80 gene panel** |  |  |  |  |  |  |
| *ACTB* | Actin, beta | - | 7p22.1 | 1852 | 102630 | Procaccio et al. (2006) |
| *ACTG1* | Actin, gamma-1 | DFNA20/26 | 17q25.3 | 1919 | 102560 | Zhu et al. (2003) |
| *ATP6V1B1* | ATPase, H+ transporting, lysosomal, 56/58-KD, V1 subunit B, isoform 1 | - | 2q13.3 | 1956 | 192132 | Karet et al. (1999) |
| *BCS1L* | BCS1, S. cerevisiae, homologue-like | - | 2q35 | 1663 | 603647 | Hinston et al. (2007) |
| *BSND* | Bartter syndrome, infantile, with sensorineural deafness (Barttin) | DFNB73 | 1q32.3 | 1396 | 606412 | Riazuddin et al. (2009) |
| *CATSPER2* | Cation channel, sperm-associated 2 | - | 15q15.3 | 1948 | 607249 | Zhang et al. (2007) |
| *CCDC50* | Coiled-coil domain-containing protein 50 | DFNA44 | 3q28 | 2454 | 611051 | Modamio-Hoybjor et al. (2003) |
| *CDH23* | Cadherin 23 | DFNB12 | 10q22.1 | 11073 | 605516 | Borck et al. (2001) |
| *CLDN14* | Claudin 14 | DFNB29 | 21q22.13 | 1943 | 605608 | Wilcox et al. (2001) |
| *COCH* | Coagulation factor C homology | DFNA9 | 14q12 | 2534 | 603196 | Robertson et al. (1998) |
| *COL11A2* | Collagen, type XI, alpha-2 | DFNA13; DFNB53 | 6p21.32 | 6414 | 120290 | McGuirt et al. (1999); Chen et al. (2005) |
| *COL9A3* | Collagen, type IX, alpha-3 | - | 20q13.33 | 2485 | 120270 | Asamura et al. (2005) |
| *CRYM* | Crystallin, mu | DFNA40 | 16q12.2 | 1303 | 123740 | Abe et al. (2003) |
| *DFNA5* | Deafness, autosomal dominant 5 | DFNA5 | 7p15.3 | 2230 | 608798 | Van Laer et al. (1998) |
| *DFNB31* | Deafness, autosomal recessive 31 | DFNB31 | 9q32 | 4022 | 607084 | Mustapha et al. (2002) |
| *DIAPH1* | Diaphanous, Drosophila, homolog 1 | DFNA1 | 5q31.3 | 5662 | 602121 | Lynch et al. (1997) |
| *DSPP* | Dentin sialophosphoprotein | DFNA39 | 4q22.1 | 4187 | 125485 | Xiao et al. (2001) |
| *ERCC2* | Excision repair, complementing defective, in Chinese hamster 2 | - | 19q13.32 | 2568 | 126340 | Flores-Alvarado et al. (2010) |
| *ERCC3* | Excision repair, complementing defective, in Chinese hamster 3 | - | 2q14.3 | 2751 | 133510 | Flores-Alvarado et al. (2010) |
| *ESPN* | Espin | DFNB36 | 1q36.31 | 3542 | 606351 | Naz et al. (2004) |
| *ESRRB* | Estrogen-related receptor, beta | DFNB35 | 14q24.3 | 2193 | 602167 | Ansar et al. (2003) |
| *EYA4* | Eyes absent 4 | DFNA10 | 6q23.2 | 3077 | 603550 | Wayne et al. (2001) |
| *FGF3* | Fibroblast growth factor 3 | - | 11q13.3 | 1548 | 164950 | Tekin et al. (2008) |
| *GATA3* | GATA-binding protein 3 | - | 10p14 | 3067 | 131320 | Van Esch et al. (2000) |
| *GJA1* | Gap junction protein, alpha-1 | DFNB38 | 6q22.31 | 3130 | 121014 | Liu et al. (2001) |
| *GJB1* | Gap junction protein, beta-1 | - | Xq13.1 | 1623 | 304040 | Bergoffen et al. (1993) |
| *GJB2* | Gap junction protein, beta-2 | DFNA3A; DFNB1A | 13q12.11 | 2263 | 121011 | Kelsell et al. (1997); Carrasquello et al. (1997) |
| *GJB3* | Gap junction protein, beta-3 | DFNA2B | 1p34.3 | 1777 | 603324 | Xia et al. (1998) |
| *GJB4* | Gap junction protein, beta-4 | - | 1p34.2 | 2840 | 605425 | Lopez-Bigas et al. (2002) |
| *GJB6* | Gap junction protein, beta-6 | DFNA3B; DFNB1B | 13q12.11 | 1805 | 604418 | Grifa et al. (1999) |
| *GRHL2* | Grainyhead-like 2 | DFNA28 | 8q22.3 | 5231 | 608576 | Peters et al. (2002) |
| *GSTP1* | Glutathione S-transferase, pi | - | 11q13.2 | 986 | 134660 | Ateş et al. (2005) |
| *JAG1* | Jagged 1 | - | 20p12.2 | 5988 | 601920 | Le Caignec et al. (2002) |
| *KCNE1* | Potassium channel, voltage-gated, Isk-related subfamily, member 1 | - | 21q22.12 | 3338 | 176261 | Van Laer et al. (2006) |
| *KCNJ10* | Potassium channel, inwardly rectifying, subfamily J, member 10 | - | 1q23.2 | 5323 | 602208 | Yang et al. (2009) |
| *KCNQ4* | Potassium channel, voltage-gated, KQT-like subfamily, member 4 | DFNA2A | 1p34.3 | 2335 | 603537 | Kubisch et al. (1999) |
| *LHFPL5* | Lipoma HMGIC fusion partner-like 5 | DFNB66/67 | 6p21.31 | 2162 | 609427 | Kalay et al. (2006) |
| *LHX3* | LIM homeobox gene 3 | - | 9p34.3 | 2376 | 600577 | Rjab et al. (2008) |
| *LRTOMT* | Leucine-rich transmembrane and O-methyltransferase | DFNB63 | 11q13.4 | 2332 | 612414 | Ahmed et al. (2008) |
| *MARVELD2* | MARVEL domain-containing protein 2 | DFNB49 | 5q13.2 | 2385 | 610572 | Riazuddin et al. (2006) |
| *MTAP* | Methylthioadenosine phosphorylase | - | 9p21.3 | 4937 | 156540 | Williamson et al. (2007) |
| *MYH14* | Myosin, heavy chain 14, nonmuscle | DFNA4A | 19q13.33 | 6786 | 608568 | Donaudy et al. (2004) |
| *MYH9* | Myosin, heavy chain 9, nonmuscle | DFNA17 | 22q12.3 | 7474 | 160775 | Lalwani et al. (2000) |
| *MYO1A* | Myosin IA | DFNA48 | 12q13.3 | 3624 | 601478 | Donaudy et al. (2003) |
| *MYO1C* | Myosin IC | - | 17p13.3 | 4973 | 606538 | Zadaro et al. (2009) |
| *MYO1F* | Myosin IF | - | 19p13.2 | 4173 | 601480 | Chen et al. (2001) |
| *MYO3A* | Myosin IIIA | DFNB30 | 10p12.1 | 5597 | 606808 | Walsh et al. (2002) |
| *MYO6* | Myosin VI | DFNA22; DFNB37 | 6q14.1 | 5278 | 600970 | Melchioda et al. (2001); Ahmed et al. (2003) |
| *MYO7A* | Myosin VIIA | DFNA11; DFNB2 | 11q13.5 | 7465 | 276903 | Liu et al. (1997) |
| *MYO15A* | Myosin XVA | DFNB3 | 17p11.2 | 11876 | 602666 | Wang et al. (1998) |
| *NR2F1* | Nuclear receptor subfamily 2, group F, member 1 | - | 5q15 | 3210 | 132890 | Brown et al. (2009) |
| *OTOA* | Otoancorin | DFNB22 | 16p12.2 | 3625 | 607038 | Zwaenepoel et al. (2002) |
| *OTOF* | Otoferlin | DFNB9 | 2p23.3 | 7173 | 603681 | Yasunaga et al. (2000) |
| *OTOR* | Otoraplin | - | 20p12.1 | 1477 | 606067 | Rendtorff et al. (2011) |
| *PAX3* | Paired box gene 3 | - | 2q36.1 | 3359 | 606597 | Baldwin et al. (1992) |
| *PCDH15* | Protocadherin 15 | DFNB23 | 10q21.1 | 7022 | 605514 | Ahmed et al. (2003) |
| *PDZD7* | PDZ domain-containing 7 | - | 10q24.31 | 2072 | 612971 | Schneider et al. (2009) |
| *PJVK* | Pejvakin | DFNB59 | 2q31.2 | 1415 | 610219 | Delmaghani et al. (2006) |
| *PMP22* | Peripheral myelin protein 22 | - | 17p12 | 1828 | 601097 | Boerkoel et al. (2002) |
| *POU3F4* | POU domain, class 3, transcription factor 4 | DFNX2 | Xq21.1 | 1491 | 300039 | De Kok et al. (1995) |
| *POU4F3* | POU domain, class 4, transcription factor 3 | DFNA15 | 5q32 | 1017 | 602460 | Vahava et al. (1998) |
| *RDX* | Radixin | DFNB24 | 11q22.3 | 4498 | 179410 | Khan et al. (2007) |
| *SLC4A11* | Solute carrier family 4 (sodium borate transporter), member 11 | - | 20p13 | 3110 | 610206 | Desir et al. (2007) |
| *SLC17A8* | Solute carrier family 17 (sodium phosphate cotransporter), member 8 | DFNA25 | 12q23.1 | 3983 | 607557 | Ruel et al. (2008) |
| *SLC26A4* | Solute carrier family 26, member 4 | DFNB4 | 7q22.3 | 4930 | 605646 | Li et al. (1998) |
| *SLC26A5* | Solute carrier family 26, member 5 | DFNB61 | 7q22.1 | 2671 | 604943 | Liu et al. (2003) |
| *SOX2* | SRY-box 2 | - | 3q26.33 | 2518 | 184429 | Kelberman et al. (2006) |
| *SPINK5* | Serine protease inhibitor, Kazal-type 5 | - | 5q32 | 3655 | 605010 | Chavanas et al. (2000) |
| *STRC* | Stereocilin | DFNB16 | 15q15.3 | 5516 | 606440 | Verpy et al. (2001) |
| *TBL1X* | Transducin-beta-like 1, X-linked | - | Xp22.3-p22.2 | 5586 | 300196 | Yan, et al. (2005) |
| *TCF21* | Transcription factor 21 | - | 6q23.2 | 3249 | 603306 | Schonberger et al. (2005) |
| *TECTA* | Tectorin, alpha | DFNA8/12; DFNB21 | 11q23.3 | 6469 | 602574 | Verhoeven et al. (1998); Mustapha et al. (1999) |
| *TIMM8A* | Translocase of inner mitochondrial membrane 8, yeast homolog A | - | Xq22.1 | 1459 | 300356 | Tranebjaerg et al. (2000) |
| *TMC1* | Transmembrane channel-like protein 1 | DFNA36; DFNB7/11 | 9q21.13 | 3201 | 606706 | Kurima et al. (2002) |
| *TMIE* | Transmembrane inner ear-expressed gene | DFNB6 | 3p21.31 | 1646 | 607237 | Naz et al. (2002) |
| *TMPRSS3* | Transmembrane protease, serine 3 | DFNB8/10 | 21q22.3 | 2468 | 605511 | Scott et al. (2001) |
| *TMPRSS5* | Transmembrane protease, serine 5 | - | 11q23.2 | 2233 | 606751 | Guipponi et al. (2008) |
| *TRIOBP* | TRIO- and F-actin binding protein | DFNB28 | 22q13.1 | 10024 | 609761 | Shahin et al. (2006) |
| *USH1C* | Usher syndrome, type 1C | DFNB18A | 11p15.1 | 2228 | 605242 | Verpy et al. (2000) |
| *WFS1* | Wolframin | DFNA6/14/38 | 4p16.1 | 3640 | 606201 | Baspalova et al. (2001) |
| **Additional genes in 129 gene panel** | | | | | | |
| *ATP6V1B2* | ATPase, H+ transporting, lysosomal, 56/58-KD, V1 subunit B, isoform 2 | - | 6p21.3 | 3054 | 606939 | Lee et al. (1995) |
| *CEACAM16* | Carcinoembryonic antigen-related cell adhesion molecule 16 | DFNA4B | 19q13.32 | 1692 | 614591 | Zheng et al. (2011) |
| *CLRN1* | Clarin 1 | - | 3q25.1 | 2359 | 606397 | Ness et al. (2003) |
| *COL9A2* | Collagen, type IX, alpha-2 | - | 1p34.2 | 2831 | 120260 | Baker et al. (2011) |
| *ECE1* | Endothelin-converting enzyme 1 | - | 1p36.12 | 5114 | 600423 | Albertin et al. (1996) |
| *EDNRA* | Endothelin receptor, type A | - | 4q31.22 | 4168 | 131243 | Tzourio et al. (2001) |
| *EDNRB* | Endothelin receptor, type B | - | 13q22.3 | 4296 | 131244 | Puffenberger et al. (1994) |
| *FAS* | Tumor necrosis factor receptor superfamily, member 6 | - | 10q23.31 | 2755 | 134637 | Rieux-Laucat et al. (1995) |
| *FGFR3* | Fibroblast growth factor receptor 3 | - | 4p16.3 | 4304 | 134934 | Toydemir et al. (2006) |
| *FOXI1* | Forkhead box I1 | - | 5q35.1 | 2296 | 601093 | Hulander et al. (1998) |
| *GIPC3* | GIPC PDZ domain-containing family, member 3 | DFNB15 | 19p13.3 | 4317 | 608792 | [Charizopoulou et al. (2011)](http://omim.org/entry/608792?search=GIPC3&highlight=gipc3#reference2) |
| *GPR98* | G protein-coupled receptor 98 | - | 5q14.3 | 19333 | 602851 | Weston et al. (2003) |
| *GPSM2* | G protein signaling modulator 2 | DFNB82 | 1p13.3 | 3039 | 609245 | Walsh et al. (2010) |
| *GRXCR1* | Glutaredoxin, cysteine-rich 1 | DFNB25 | 4p13 | 1003 | 613283 | Schraders et al. (2010) |
| *HAL* | Histadine ammonia-lyase | - | 12q23.1 | 3927 | 609457 | Kawai et al. (2005) |
| *HGF* | Hepatocyte growth factor | DFNB39 | 7q21.11 | 2820 | 142409 | Schultz et al. (2009) |
| *ILDR1* | Immunoglobulin-like domain-containing receptor 1 | DFNB42 | 3q13.33 | 2908 | 609739 | Borck et al. (2011) |
| *KCNQ1* | Potassium channel, voltage-gated, KQT-like subfamily, member 1 | - | 11p15.5 | 3262 | 607542 | Reardon et al. (1993) |
| *KIAA1199* | KIAA1199 protein | - | 15q25.1 | 7080 | 608366 | Abe et al. (2003) |
| *LOXHD1* | Lipoxygenase homology domain-containing 1 | DFNB77 | 18q21.1 | 6854 | 613072 | Grillet et al. (2009) |
| *MIR96* | Micro RNA 96 | DFNA50 | 7q32.2 | 78 | 611606 | Mencia et al. (2010) |
| *MIR182* | Micro RNA 182 | - | 7q32.2 | 110 | 611607 | Weston et al. (2006) |
| *MIR183* | Micro RNA 183 | - | 7q32.2 | 110 | 611608 | Weston et al. (2006) |
| *MITF* | Microphthalmia-associated transcription factor | - | 3p13 | 4815 | 156845 | Tassabehji et al. (1994) |
| *MSRB3* | Methionine sulfoxide reductase B3 | DFNB74 | 12q14.3 | 4307 | 613719 | Waryah et al. (2009) |
| *MT-TD* | Transfer RNA, mitochondrial, aspartic acid | - | Mito. | 68 | 590015 | Seneca et al. (2005) |
| *MT-TH* | Transfer RNA, mitochondrial, histidine | - | Mito. | 69 | 590040 | Crimi et al. (2003) |
| *MT-TI* | Transfer RNA, mitochondrial, isoleucine | - | Mito. | 69 | 590045 | Corona et al. (2002) |
| *MT-TK* | Transfer RNA, mitochondrial, lysine | - | Mito. | 70 | 590060 | Silvestri et al. (1992) |
| *MT-TL1* | Transfer RNA, mitochondrial, leucine 1 | - | Mito. | 75 | 590050 | Mosewich et al. (1993) |
| *MT-TL2* | Transfer RNA, mitochondrial, leucine 2 | - | Mito. | 71 | 590055 | Fu et al. (1996) |
| *MT-TM* | Transfer RNA, mitochondrial, methionine | - | Mito. | 68 | 590065 | Jones et al. (2008) |
| *MT-TQ* | Transfer RNA, mitochondrial, glutamine | - | Mito. | 72 | 590030 | Finnila et al. (2001) |
| *MT-TS1* | Transfer RNA, mitochondrial, serine 1 | - | Mito. | 69 | 590080 | Reid et al. (1994) |
| *MT-TS2* | Transfer RNA, mitochondrial, serine 2 | - | Mito. | 59 | 590085 | Mansergh et al. (1999) |
| *NDP* | Norrin | - | Xp11.3 | 2058 | 300658 | Rehm et al. (1997) |
| *P2RX2* | Purinergin receptor P2X, ligand-gated ion channel 2 | - | 12q24.33 | 1945 | 600844 | Yan et al. (2013) |
| *PRPS1* | Phosphoribosylpyrophosphate synthetase 1 | DFNX1 | Xq22.3 | 2156 | 311850 | Liu et al. (2010) |
| *PTPRQ* | Protein-tyrosine phosphatase receptor-type Q | DFNB84A | 12q21.31 | 8066 | 603317 | Schraders et al. (2013) |
| *SERPINB6* | Serpin peptidase inhibitor, clade B (ovalbumin), member 6 | DFNB91 | 6p25.2 | 1932 | 173321 | Sirmanci et al. (2010) |
| *SIX1* | Sine oculis homeobox, drosophila, homolog 1 | DFNA23 | 14q23.1 | 2687 | 601205 | Ruf et al. (2004) |
| *SIX5* | Sine oculis homeobox, drosophila, homolog 5 | - | 19q13.32 | 3352 | 600963 | Hoskins et al. (2007) |
| *SMPX* | Small muscle protein, X-linked | DFNX4 | Xp22.12 | 951 | 300226 | Schraders et al. (2011) |
| *SNAI2* | Snail, drosophila, homolog 2 | - | 8q11.21 | 2112 | 602150 | Sanchez-Martin et al. (2002) |
| *TFCP2* | Transcription factor CP2 | - | 12q13.12-q13.13 | 3715 | 189889 | Swendeman et al. (1994) |
| *TJP2* | Tight junction protein 2 | DFNA51 | 9q21.11 | 4725 | 607709 | Walsh et al. (2010) |
| *TPRN* | Taperin | DFNB79 | 9q34.3 | 2641 | 613354 | Rehman et al. (2010) |
| *USH1G* | Usher syndrome 1G | - | 17q25.1 | 3568 | 607696 | Bashir et al. (2010) |
| *USH2A* | Usher syndrome 2A | - | 1q41 | 18883 | 608400 | Van Wijk et al. (2004) |
