# Supplementary material for: Targeted next-generation sequencing of deafness genes in hearing-impaired individuals uncovers informative mutations
Source: Genet Med. 2014 May 29;16(12):945–53. doi: 10.1038/gim.2014.65 (PMC4262760; doi:10.1038/gim.2014.65)
Supplement: Supplementary Table S3 [file gim201465x4.doc]

**Table S3** Run statistics and target coverage information from human deafness gene panel sequencing

| **Individual** | **Number of reads*** | **Mapped reads*** | **Mean depth (x)** | **% of exons with Depth ≥10x** |
| --- | --- | --- | --- | --- |
| D1^a^ | 6,325,660 | 4,815,197 | 176 | 95.4 |
| D2^a^ | 13,618,872 | 11,778,140 | 409 | 99.7 |
| D3^a^ | 7,318,278 | 5,560,271 | 197 | 95.7 |
| D4^a^ (I:4) | 10,945,026 | 9,427,871 | 309 | 99.5 |
| D4^a^ (II:6) | 12,107,818 | 10,763,523 | 345 | 99.6 |
| D5^b^ | 6,313,548 | 5,797,322 | 242 | 99.4 |
| D6^a^ | 5,318,288 | 4,909,669 | 396 | 99.6 |
| D7^b^ | 7,463,506 | 6,822,238 | 282 | 99.6 |
| D8^a^ (III:2) | 5,741,568 | 5,215,973 | 437 | 99.5 |
| D8^a^ (IV:2) | 5,019,068 | 4,556,298 | 352 | 99.5 |
| R1^a^ | 4,792,488 | 4,410,058 | 377 | 99.7 |
| R2^a^ (VII:4) | 5,479,774 | 4,211,942 | 147 | 95.4 |
| R2^a^ (V:8) | 6,768,524 | 5,029,245 | 177 | 95.5 |
| R3^b^ | 7,314,120 | 6,658,222 | 284 | 99.7 |
| R4^b^ | 6,814,618 | 6,202,347 | 261 | 99.5 |
| R5^b^ (II:2) | 6,931,570 | 6,438,225 | 204 | 92.5 |
| U1^a^ | 7,561,986 | 5,752,732 | 207 | 95.8 |
| U2^a^ | 7,114,584 | 5,376,162 | 195 | 95.6 |
| U3^a^ | 5,131,806 | 4,716,270 | 408 | 99.7 |
| U4A^b^ | 6,331,630 | 5,843,843 | 198 | 99.1 |
| U4B^b^ | 6,916,206 | 6,378,414 | 230 | 99.3 |
| U5A^a^ | 4,635,632 | 4,270,323 | 326 | 99.5 |
| U5B^a^ | 4,356,056 | 3,972,174 | 318 | 99.6 |
| U6A^a^ | 14,706,372 | 13,247,035 | 474 | 99.5 |
| U6B^a^ | 12,850,352 | 11,452,294 | 410 | 99.5 |
| U7^a^ | 7,377,024 | 5,536,805 | 199 | 99.4 |
| U8^b^ | 6,092,050 | 5,585,863 | 252 | 99.4 |
| U9A^a^ | 6,858,184 | 5,198,277 | 181 | 95.5 |
| U9B^a^ | 6,791,602 | 5,211,423 | 195 | 95.8 |
| U10^a^ | 7,142,816 | 5,156,676 | 191 | 95.6 |
| Control 1^a^ | 13,844,186 | 12,470,109 | 447 | 99.5 |
| Control 2^a^ | 14,257,838 | 12,318,824 | 433 | 99.5 |
| Control 3^a^ | 10,985,596 | 9,618,643 | 322 | 99.5 |
| Control 4^a^ | 12,704,104 | 11,408,390 | 408 | 99.5 |
| Control 5^a^ | 12,590,204 | 11,087,703 | 375 | 99.5 |
| Control 6^a^ | 3,875,190 | 3,589,027 | 280 | 99.5 |
| Control 7^a^ | 4,680,476 | 4,295,677 | 366 | 99.7 |
| Control 8^a^ | 4,211,518 | 3,891,516 | 298 | 99.5 |
| Control 9^b^ | 6,971,952 | 6,493,175 | 263 | 99.5 |

*Number of reads and number of mapped reads are in bp.

^a^80 gene panel; ^b^129 gene panel.
