# Supplementary material for: Targeted next-generation sequencing of deafness genes in hearing-impaired individuals uncovers informative mutations
Source: Genet Med. 2014 May 29;16(12):945–53. doi: 10.1038/gim.2014.65 (PMC4262760; doi:10.1038/gim.2014.65)
Supplement: Supplementary Table S4 [file gim201465x5.doc]

**Table S4** Summary of all damaging variants and mutations per patient according to group

| **Cases** | **Gene^1^** | **DFN Locus** | **Nucleotide** | **Protein** | **Mutat.Taster^2^** | **PolyPhen2^2^** | **SIFT^3^** | **Zyg.^4^** | **Depth** | **MAF** | **dbSNP** |
| --- | --- | --- | --- | --- | --- | --- | --- | --- | --- | --- | --- |
| **Patients with dominant forms of deafness** | | | | | | | | | | | |
| D1 | ***MYO6*** | DFNA22 | c.884_893delGCAAAAGTCC | p.Arg295Leufs*13 | DC (1.0) | NA | NA | 0/1 | 44 | - | - |
| D1 | *DSPP* | DFNA39 | c.691C>A | p.Pro231Thr | B (1.0) | PoD (0.90) | D (0.00) | 0/1 | 306 | A = 0.010 | rs61738509 |
| D1 | *DSPP* | DFNA39 | c.802G>T | p.Gly268Trp | B (1.0) | PrD (1.0) | D (0.00) | 0/1 | 298 | T = 0.010 | rs61738508 |
| D1 | *KCNJ10* | - | c.811C>T | p.Arg271Cys | DC (1.0) | PrD (1.0) | D (0.02) | 0/1 | 287 | A = 0.023 | rs1130183 |
| D1 | *OTOF* | DFNB9 | c.1393-1G>C | - | NA | NA | NA | 0/1 | 208 | - | - |
| D1 | *OTOF* | DFNB9 | c.5558G>A | p.Arg1853Gln | DC (1.0) | PrD (0.98) | D (0.00) | 0/1 | 80 | T = 0.006 | rs111033329 |
| D1 | *TRIOBP* | DFNB28 | c.3089C>G | p.Pro1030Arg | B (0.96) | PrD (1.0) | D (0.00) | 0/1 | 110 | G = 0.005 | rs193013234 |
| D2 | ***ACTG1*** | DFNA20/26 | c.974T>A | p.Met325Lys | DC (1.0) | B (0.00) | D (0.00) | 0/1 | 181 | - | - |
| D2 | *ESPN* | DFNB36 | c.752G>A | p.Gly251Asp | DC (1.0) | PrD (1.0) | B (0.29) | 0/1 | 354 | - | rs200602012 |
| D2 | *SLC26A4* | DFNB4 | c.2326C>T | p.Arg776Cys | DC (1.0) | PrD (1.0) | D (0.01) | 0/1 | 183 | T = 0.001 | rs111033255 |
| D2 | *MYO1C* | - | c.859G>A | p.Val287Ile | DC (1.0) | PrD (0.98) | B (0.05) | 0/1 | 143 | T = 0.001 | rs117696188 |
| D2 | *USH1C* | DFNB18 | c.1823C>G | p.Pro608Arg | DC (0.98) | NA | B (1.00) | 0/1 | 212 | - | rs41282932 |
| D3 | ***TCF21*** | - | c.63C>G | p.Asp21Glu | DC (1.0) | B (0.15) | D (0.00) | 0/1 | 374 | G = 0.006 | rs61729591 |
| D3 | *CDH23* | DFNB12 | c.2001C>A | p.Asn667Lys | DC (1.0) | PrD (1.0) | D (0.00) | 0/1 | 10 | - | - |
| D3 | *OTOR* | - | c.2T>C | p.Met1Thr | DC (1.0) | PoD (0.96) | D (0.00) | 0/1 | 108 | C = 0.029 | rs17686437 |
| D4 | ***CCDC50*** | DFNA44 | c.227G>A | p.Arg76His | DC (0.99) | B (0.21) | D (0.01) | 0/1 | 80 | A = 0.001 | rs138443787 |
| D4 | *CCDC50* | DFNA44 | c.157C>T | p.Gln53* | DC (1.0) | NA | NA | 0/1 | 107 | - | - |
| D4 | *GATA3* | - | c.231_238delGACCCACC | p.Thr78Argfs*223 | DC (1.0) | NA | NA | 0/1 | 202 | - | - |
| D4 | *MYO1C* | - | c.319G>A | p.Val107Ile | DC (1.0) | PoD (0.62) | B (0.28) | 0/1 | 101 | T = 0.001 | rs140549082 |
| D4 | *SPINK5* | - | c.3134G>A | p.Gly1045Asp | DC (0.99) | PrD (1.0) | D (0.00) | 0/1 | 309 | - | - |
| D5 | ***MYO1A*** | DFNA48 | c.2032A>T | p.Ile678Phe | DC (1.0) | PrD (0.99) | D (0.00) | 0/1 | 46 | A = 0.003 | rs151269703 |
| D5 | *DSPP* | DFNA39 | c.974G>C | p.Ser325Thr | B (0.99) | PrD (0.97) | D (0.00) | 0/1 | 315 | C = 0.001 | rs145481275 |
| D5 | *OTOR* | - | c.2T>C | p.Met1Thr | DC (1.0) | PoD (0.96) | D (0.00) | 0/1 | 176 | C = 0.029 | rs17686437 |
| D6 | ***MYH14*** | DFNA4A | c.5008C>T | p.Arg1670Cys | DC (1.0) | PrD (1.0) | D (0.00) | 0/1 | 330 | - | - |
| D6 | *CCDC50* | DFNA44 | c.1396C>T | p.Arg466Trp | B (1.0) | NA | D (0.05) | 0/1 | 75 | T = 0.006 | rs147604673 |
| D6 | *GJB4* | - | c.384G>A | p.Trp128* | DC (1.0) | NA | NA | 0/1 | 398 | A = 0.002 | rs149110828 |
| D7 | ***MYO1A*** | DFNA48 | c.2390C>T | p.Ser797Phe | DC (0.89) | PrD (0.71) | D (0.00) | 0/1 | 73 | A = 0.003 | rs113470661 |
| D7 | *MYH14* | DFNA4A | c.483G>A | p.Met161Ile | DC (1.0) | PoD (0.76) | D (0.00) | 1/1 | 351 | A = 0.013 | rs34773557 |
| D7 | *LHFPL5* | DFNB67 | c.169C>T | p.Pro57Ser | DC (1.0) | PrD (1.0) | B (0.28) | 0/1 | 380 | - | - |
| D8 | ***EYA4*** | DFNA10 | c.1341-19T>A | - | NA | NA | NA | 0/1 | 351 | - | - |
| D8 | *CDH23* | DFNB12 | c.1096G>A | p.Ala366Thr | DC (1.0) | PrD (1.0) | D (0.00) | 0/1 | 255 | A = 0.004 | rs143282422 |
| D8 | *GJB3* | DFNB91 | c.94C>T | p.Arg32Trp | DC (1.0) | PrD (1.0) | D (0.00) | 0/1 | 401 | T = 0.015 | rs1805063 |
| D8 | *GJB4* | - | c.155_158delTCTG | p.Val52Alafs*55 | DC (1.0) | NA | NA | 0/1 | 333 | - | - |
| D8 | *MYH9* | DFNA17 | c.193G>A | p.Val65Met | DC (0.93) | B (0.37) | D (0.01) | 0/1 | 257 | - | - |
| D8 | *MYO3A* | DFNB30 | c.2387T>G | p.Phe796Cys | DC (1.0) | PrD (1.0) | D (0.00) | 0/1 | 36 | - | - |
| D8 | *TECTA* | DFNB21 | c.4355T>A | p.Phe1452Tyr | DC (0.98) | PrD (0.99) | B (0.11) | 0/1 | 172 | - | - |
| **Patients with recessive forms of deafness** | | | | | | | | | | | |
| R1 | ***MYO15A*** | DFNB3 | c.1137delC | p.Tyr380Metfs*65 | DC (1.0) | NA | NA | 0/1 | 306 | - | - |
| R1 | ***MYO15A*** | DFNB3 | c.7124_7217delACAG | p.Asp2375Valfs*29 | DC (1.0) | NA | NA | 0/1 | 89 | - | - |
| R1 | *MYH9* | DFNA17 | c.7C>G | p.Gln3Glu | DC (0.98) | PoD (0.53) | D (0.00) | 0/1 | 315 | C = 0.001 | rs56200894 |
| R2 | ***MYO7A*** | DFNB2 | c.3935T>C | p.Leu1312Pro | DC (1.0) | PrD (1.0) | D (0.00) | 1/1 | 87 | - | - |
| R2 | *TMIE* | DFNB6 | c.191C>T | p.Ser64Leu | DC (1.0) | PrD (0.99) | D (0.02) | 0/1 | 183 | T = 0.001 | rs189895472 |
| R3 | ***USH2A*** | - | c.1841-2A>G | - | NA | NA | NA | 0/1 | 393 | - | - |
| R3 | ***USH2A*** | - | c.2440C>T | p.Gln814* | NA | NA | NA | 0/1 | 144 | - | - |
| R3 | *MYH14* | DFNA4A | c.1150G>T | p.Gly384Cys | DC (1.0) | PrD (1.0) | D (0.00) | 0/1 | 389 | T = 0.003 | rs119103280 |
| R3 | *MYH14* | DFNA4A | c.3805C>T | p.Gln1269* | DC (1.0) | NA | NA | 1/1 | 342 | - | - |
| R3 | *GJB2* | DFNB1A | c.35delG | p.Gly12Valfs*2 | DC (1.0) | NA | NA | 0/1 | 429 | - | rs80338939 |
| R3 | *TMPRSS5* | - | c.393G>A | p.Trp131* | DC (1.0) | NA | NA | 0/1 | 240 | - | - |
| R3 | *TRIOBP* | DFNB28 | c.1617_1619delCTC | p.Ser540del | DC (0.61) | NA | NA | 0/1 | 331 | - | - |
| R4 | ***USH2A*** | - | c.2276G>T | p.Cys759Phe | DC (1.0) | PrD (1.0) | D (0.00) | 0/1 | 130 | A = 0.001 | rs80338902 |
| R4 | *MARVELD2* | DFNB49 | c.466C>T | p.Arg156Trp | B (1.0) | PrD (0.97) | D (0.05) | 0/1 | 128 | - | rs142099889 |
| R4 | *MYH14* | DFNA4A | c.483G>A | p.Met161Ile | DC (1.0) | PoD (0.76) | D (0.00) | 0/1 | 349 | A = 0.013 | rs34773557 |
| R5 | ***GJB2*** | DFNB1A | c.35delG | p.Gly12Valfs*2 | DC (1.0) | NA | NA | 1/1 | 205 | - | rs80338939 |
| R5 | *ERCC2* | - | c.1381C>G | p.Leu461Val | DC (1.0) | PrD (0.98) | B (0.13) | 0/1 | 53 | - | rs121913016 |
| R5 | *TECTA* | DFNB12 | c.5012C>T | p.Ser1671Leu | DC (1.0) | B (0.172) | D (0.05) | 0/1 | 189 | T = 0.001 | rs142948530 |
| R5 | *USH2A* | - | c.1663C>G | p.Leu555Val | DC (1.0) | PrD (1.0) | D (0.00) | 0/1 | 217 | C = 0.001 | rs35818432 |
| **Unsolved cases** | | | | | | | | | | | |
| U2 | *GJB4* | - | c.155_158delTCTG | p.Val52Alafs*55 | DC (1.0) | NA | NA | 0/1 | 468 | - | - |
| U2 | *MYO1A* | DFNA48 | c.1985G>A | p.Gly662Glu | DC (0.10) | B (0.00) | D (0.04) | 0/1 | 89 | T = 0.015 | rs33962952 |
| U2 | *PCDH15* | DFNB23 | c.2252C>A | p.Ala751Asp | DC (1.0) | PrD (0.98) | D (0.02) | 0/1 | 121 | - | - |
| U2 | *SLC26A4* | DFNA4 | c.760A>G | p.Ile254Val | DC (0.70) | PoD (0.94) | B (0.11) | 0/1 | 128 | - | - |
| U2 | *TMPRSS5* | - | c.1187G>A | p.Gly396Glu | DC (1.0) | PrD (1.0) | D (0.00) | 0/1 | 10 | - | - |
| U3 | *CCDC50* | DFNA44 | c.1396C>T | p.Arg466Trp | B (1.0) | NA | D (0.05) | 0/1 | 86 | T = 0.006 | rs147604673 |
| U3 | *COL9A3* | - | c.753C>G | p.Phe251Leu | DC (0.99) | PoD (0.96) | B (0.12) | 0/1 | 346 | - | - |
| U3 | *MYH9* | DFNA17 | c.5143G>A | p.Gly1715Ser | DC (0.99) | B (0.02) | D (0.04) | 0/1 | 108 | T = 0.001 | rs148109368 |
| U4 | *ESPN* | DFNB36 | c.786C>G | p.His262Gln | DC (0.98) | PoD (0.47) | B (0.41) | 0/1 | 25 | - | - |
| U4 | *MYO7A* | DFNB2 | c.2476G>T | p.Ala826Ser | DC (0.98) | B (0.15) | D (0.05) | 0/1 | 22 | - | - |
| U5 | *SLC26A4* | DFNB4 | c.1003T>C | p.Phe335Leu | DC (1.0) | PrD (1.0) | D (0.00) | 0/1 | 130 | C = 0.001 | rs111033212 |
| U5 | *OTOF* | DFNB9 | c.2464C>T | p.Arg822Trp | DC (0.98) | PrD (1.0) | D (0.00) | 0/1 | 99 | A = 0.009 | rs80356570 |
| U5 | *OTOF* | DFNB9 | c.3247G>C | p.Ala1083Pro | DC (0.60) | PoD (0.62) | D (0.04) | 0/1 | 76 | G = 0.009 | rs80356574 |
| U5 | *WFS1* | DFNA6/14/38 | c.2393_2394insACG | p.Val798_Thr799insArg | DC (0.67) | NA | NA | 0/1 | 74 | - | - |
| U6 | *ERCC2* | - | c.1726G>A | p.Glu576Lys | DC (1.0) | PrD (1.0) | D (0.00) | 0/1 | 148 | T = 0.001 | rs201165309 |
| U6 | *MYO7A* | DFNB2 | c.5227C>T | p.Arg1743Trp | DC (0.99) | PoD (0.91) | D (0.01) | 0/1 | 151 | - | rs111033287 |
| U6 | *USH1C* | DFNB18 | c.1591C>T | p.Arg531Cys | DC (0.99) | PrD (1.0) | B (0.08) | 0/1 | 219 | - | rs140528164 |
| U7 | *GJB2* | DFNB1A | c.457G>A | p.Val153Ile | DC (0.82) | B (0.00) | D (0.05) | 0/1 | 346 | T = 0.002 | rs111033186 |
| U7 | *MYO15A* | DFNB3 | c.7885G>A | p.Gly2629Ser | DC (0.56) | PoD (0.90) | B (0.09) | 0/1 | 140 | - | - |
| U7 | *TECTA* | DFNB21 | c.764A>C | p.Asp255Ala | DC (0.99) | PoD (0.94) | D (0.00) | 0/1 | 203 | - | - |
| U8 | *GJB3* | DFNB91 | c.94C>T | p.Arg32Trp | DC (1.0) | PrD (1.0) | D (0.00) | 0/1 | 337 | T = 0.015 | rs1805063 |
| U8 | *GJB4* | - | c.155_158delTCTG | p.Val52Alafs*55 | DC (1.0) | NA | NA | 0/1 | 325 | - | - |
| U8 | *GPR98* | - | c.1849G>A | p.Val617Met | DC (0.87) | PrD (1.0) | D (0.03) | 0/1 | 226 | A = 0.001 | rs199988872 |
| U8 | *GPR98* | - | c.6994A>T | p.Ile2332Phe | DC (1.0) | PrD (0.99) | D (0.02) | 0/1 | 284 | T = 0.001 | rs193030567 |
| U8 | *MYO15A* | DFNB3 | c.8782G>A | p.Asp2928Asn | DC (1.0) | PrD (1.0) | D (0.00) | 0/1 | 100 | - | - |
| U8 | *P2RX2* | DFNA41 | c.118C>T | p.Arg40Cys | DC (1.0) | PrD (1.0) | D (0.00) | 0/1 | 103 | - | - |
| U8 | *TJP2* | DFNA51 | c.116C>T | p.Thr39Met | DC (1.0) | PoD (0.74) | D (0.05) | 0/1 | 226 | T = 0.002 | rs138241615 |
| U9 | *MYH14* | DFNA4A | c.483G>A | p.Met161Ile | DC (1.0) | PoD (0.76) | D (0.00) | 0/1 | 159 | A = 0.013 | rs34773557 |
| U9 | *MYO1A* | DFNA48 | c.1985G>A | p.Gly662Glu | DC (0.10) | B (0.00) | D (0.04) | 0/1 | 84 | T = 0.015 | rs33962952 |
| U9 | *SLC26A4* | DFNB4 | c.1766A>C | p.Gln589Pro | DC (1.0) | B (0.41) | D (0.03) | 0/1 | 134 | - | - |
| U9 | *TRIOBP* | DFNB28 | c.1617_1619delCTC | p.Ser540del | DC (0.61) | NA | NA | 0/1 | 508 | - | rs146565844 |
| U10 | *OTOA* | DFNB22 | c.970A>C | p.Thr324Pro | DC (1.0) | PrD (1.0) | D (0.02) | 0/1 | 106 | - | - |
| U10 | *OTOF* | DFNB9 | c.3572A>C | p.Asp1191Ala | DC (1.0) | PrD (1.0) | D (0.00) | 0/1 | 95 | - | - |
| U10 | *PCDH15* | DFNB23 | c.5308_5315delGCTCCTCT | p.Ala1770Cysfs*5 | DC (1.0) | NA | NA | 0/1 | 179 | - | - |
| **Controls** | | | | | | | | | | | |
| 1 | *MYO1C* | - | c.2596G>A | p.Glu866Lys | DC (1.0) | PoD (0.62) | D (0.00) | 0/1 | 115 | T = 0.005 | rs61753655 |
| 2 | *MYO7A* | DFNA11/ DFNB2 | c.1960C>T | p.Arg654Cys | DC (1.0) | PrD (1.0) | D (0.00) | 0/1 | 96 | - | rs201928014 |
| 2 | *TRIOBP* | DFNB28 | c.6736G>A | p.Glu2246Lys | DC (1.0) | PrD (1.0) | D (0.00) | 0/1 | 71 | A = 0.005 | rs138139146 |
| 3 | *PCDH15* | DFNB23 | c.3832C>A | p.Gln1278Lys | DC (1.0) | PrD (0.99) | B (0.1) | 0/1 | 248 | - | - |
| 3 | *TMC1* | DFNA36/ DFNB7 | c.1141T>A | p.Tyr381Asn | DC (1.0) | PrD (1.0) | B (0.46) | 0/1 | 152 | T = 0.001 | rs111033363 |
| 4 | *CDH23* | DFBN23 | c.2239C>T | p.Arg747Cys | DC (1.0) | PrD (1.0) | D (0.00) | 0/1 | 233 | - | rs200649500 |
| 4 | *SPINK5* | - | c.1322G>A | p.Arg441His | B (1.0) | PoD (0.87) | D (0.00) | 0/1 | 367 | A = 0.004 | rs34393923 |
| 4 | *SPINK5* | - | c.2094_2096delTGG | p.Gly699del | DC (1.0) | NA | NA | 0/1 | 430 | - | rs111662216 |
| 5 | *MYO1F* | - | c.3080G>A | p.Arg1038Gln | DC (1.0) | PrD (0.99) | B (0.34) | 0/1 | 103 | - | rs200864651 |
| 6 | *WFS1* | DFNA6/14/38 | c.1597C>T | p.Pro533Ser | DC (1.0) | PrD (1.0) | D (0.00) | 0/1 | 126 | T = 0.001 | rs146132083 |
| 7 | *CDH23* | DFNB12 | c.3986G>A | p.Gly1329Asp | DC (0.98) | PrD (1.0) | D (0.00) | 0/1 | 265 | - | rs201877610 |
| 9 | *GJB3* | DFNA2B/ DFNB2B | c.94C>T | p.Arg32Trp | DC (1.0) | PrD (1.0) | D (0.00) | 0/1 | 236 | T = 0.015 | rs1805063 |
| 9 | *GJB4* | - | c.155_158delTCTG | p.Val52Alafs*55 | DC (1.0) | NA | NA | 0/1 | 196 | - | - |
| 9 | *KIAA1199* | - | c.4078_4080AAG | p.Lys1360del | DC (0.83) | NA | NA | 0/1 | 185 | T = 0.009 | rs200201338 |

^1^The quality score of all analyzed sequences was 35, which is the highest quality. The gene underlying hearing impairment in a given patient is presented in bold.

^2^MutationTaster and PolyPhen-2 operate on a scale from 0 to 1.0, with 1.0 having the highest probability of being a damaging substitution. DC=disease-causing; B=benign; NA=not analyzed; PoD=possibly damaging; PrD, probably damaging.

^3^SIFT values <0.05 predict substitutions that are deleterious (D), whereas values >0.05 predict benign (B) substitutions.

^4^Zyg, Zygosity; 1/1, homozygous, 0/1, heterozygous.
