# Supplementary material for: Targeted next-generation sequencing of deafness genes in hearing-impaired individuals uncovers informative mutations
Source: Genet Med. 2014 May 29;16(12):945–53. doi: 10.1038/gim.2014.65 (PMC4262760; doi:10.1038/gim.2014.65)
Supplement: Supplementary Table S5 [file gim201465x6.doc]

**Table S5** Variant counts of patients and controls

|  | **In silico predicted pathogenic mutations***** | | | | | |
| --- | --- | --- | --- | --- | --- | --- |
|  | **Total number** | **Dominant** | **Recessive** | **Dominant and/or recessive** | **Syndromic** | **Candidate genes** |
| **Patients with dominant HL** | | | | | | |
| D1 | 7 | 3 | 3 | 0 | 0 | 1 |
| D2 | 5 | 1 | 3 | 0 | 0 | 1 |
| D3 | 3 | 1 | 1 | 0 | 0 | 1 |
| D4 (I:4) | 5 | 2 | 0 | 0 | 2 | 1 |
| D5 | 3 | 2 | 0 | 0 | 0 | 1 |
| D6 | 3 | 2 | 0 | 0 | 0 | 1 |
| D7 | 3 | 2 | 1 | 0 | 0 | 0 |
| D8 (III:2) | 7 | 2 | 4 | 0 | 0 | 1 |
| All | 36 | 15 (42%) | 12 (33%) | 0 | 2 (6%) | 7 (19%) |
| **Patients with recessive HL** | | | | | | |
| R1 | 3 | 1 | 2 | 0 | 0 | 0 |
| R2 (VII:4) | 3 | 0 | 3 | 0 | 0 | 0 |
| R3 | 6 | 3 | 2 | 0 | 0 | 1 |
| R4 | 2 | 1 | 1 | 0 | 0 | 0 |
| R5 | 4 | 0 | 3 | 0 | 1 | 0 |
| All | 18 | 5 (28%) | 11 (61%) | 0 | 1 (6%) | 1 (6%) |
| **Unsolved cases** | | | | | | |
| U1 | 0 | 0 | 0 | 0 | 0 | 0 |
| U2 | 5 | 1 | 2 | 0 | 0 | 2 |
| U3 | 3 | 2 | 0 | 0 | 0 | 1 |
| U4 | 2 | 0 | 2 | 0 | 0 | 0 |
| U5 | 4 | 1 | 3 | 0 | 0 | 0 |
| U6 | 3 | 0 | 2 | 0 | 1 | 0 |
| U7 | 3 | 0 | 3 | 0 | 0 | 0 |
| U8 | 3 | 0 | 2 | 0 | 0 | 1 |
| U9 | 4 | 2 | 2 | 0 | 0 | 0 |
| U10 | 3 | 0 | 3 | 0 | 0 | 0 |
| All | 30 | 6 (20%) | 19 (63%) | 0 | 1 (3%) | 4 (13%) |
| **Controls without HL** | | | | | | |
| C1 | 1 | 0 | 0 | 0 | 0 | 1 |
| C2 | 2 | 0 | 2 | 0 | 0 | 0 |
| C3 | 2 | 0 | 1 | 1 | 0 | 0 |
| C4 | 3 | 0 | 1 | 0 | 2 | 0 |
| C5 | 1 | 0 | 0 | 0 | 0 | 1 |
| C6 | 1 | 1 | 0 | 0 | 0 | 0 |
| C7 | 1 | 0 | 1 | 0 | 0 | 0 |
| C8 | 0 | 0 | 0 | 0 | 0 | 0 |
| C9 | 2 | 0 | 1 | 0 | 0 | 1 |
| All | 13 | 1 (8%) | 6 (46%) | 1 (8%) | 2 (15%) | 3 (23%) |

*Patients with the 129 gene panel were reduced to the same overlapping 80 genes contained in the 80 gene panel.
